# RENaBack: low back pain patients in rehabilitation—study protocol for a multicenter, randomized controlled trial

**DOI:** 10.1186/s13063-021-05823-3

**Published:** 2021-12-18

**Authors:** Laura Puerto Valencia, Diamantes Arampatzis, Heidrun Beck, Karsten Dreinhöfer, David Drießlein, Wilfried Mau, Julia-Marie Zimmer, Michael Schäfer, Friedemann Steinfeldt, Pia-Maria Wippert

**Affiliations:** 1 Medical Sociology and Psychobiology, University of Potsdam, Potsdam, Germany; 2 Department of Training and Movement Sciences, Humboldt-Universität zu Berlin, Berlin, Germany; 3 University Center for Orthopaedics and Trauma Surgery, Hospital Carl Gustav Carus at Technical University Dresden, Dresden, Germany; 4 Statistical Consulting Unit StaBLab, Ludwig-Maximilians-Universität München, Munich, Germany; 5 Department of Orthopaedics and Trauma Surgery, Medical Park Berlin Humboldtmühle, Charité Berlin, Germany; 6 Institute for Rehabilitation Medicine, Interdisciplinary Center for Health Sciences, Medical Faculty, Martin-Luther-University (MLU) Halle-Wittenberg, Halle, Germany; 7 Department of Orthopedics, German Pension Insurance Berlin-Brandenburg, Rehabilitation Clinic Hohenelse, Rheinsberg, Germany; 8 Johannesbad Specialist Clinic & Health Center Raupennest, Orthopedic Clinic in Altenberg, Altenberg, Germany; 9 Faculty of Health Sciences Brandenburg, University of Potsdam, Am Neuen Palais 10, 14469 Potsdam, Germany

**Keywords:** Chronic low back pain, Aftercare, Individualized therapy, Randomized controlled trial, Rehabilitation

## Abstract

**Background:**

Millions of people in Germany suffer from chronic pain, in which course and intensity are multifactorial. Besides physical injuries, certain psychosocial risk factors are involved in the disease process. The national health care guidelines for the diagnosis and treatment of non-specific low back pain recommend the screening of psychosocial risk factors as early as possible, to be able to adapt the therapy to patient needs (e.g., unimodal or multimodal). However, such a procedure has been difficult to implement in practice and has not yet been integrated into the rehabilitation care structures across the country.

**Methods:**

The aim of this study is to implement an individualized therapy and aftercare program within the rehabilitation offer of the German Pension Insurance in the area of orthopedics and to examine its success and sustainability in comparison to the previous standard aftercare program.

The study is a multicenter randomized controlled trial including 1204 patients from six orthopedic rehabilitation clinics. A 2:1 allocation ratio to intervention (individualized and home-based rehabilitation aftercare) versus the control group (regular outpatient rehabilitation aftercare) is set. Upon admission to the rehabilitation clinic, participants in the intervention group will be screened according to their psychosocial risk profile. They could then receive either unimodal or multimodal, together with an individualized training program. The program is instructed in the clinic (approximately 3 weeks) and will continue independently at home afterwards for 3 months. The success of the program is examined by means of a total of four surveys. The co-primary outcomes are the Characteristic Pain Intensity and Disability Score assessed by the German version of the Chronic Pain Grade questionnaire (CPG).

**Discussion:**

An improvement in terms of pain, work ability, patient compliance, and acceptance in our intervention program compared to the standard aftercare is expected. The study contributes to provide individualized care also to patients living far away from clinical centers.

**Trial registration:**

DRKS, DRKS00020373. Registered on 15 April 2020

**Supplementary Information:**

The online version contains supplementary material available at 10.1186/s13063-021-05823-3.

## Administrative information

**Table:** 

Title {1}	RENaBack: Low back pain patients in rehabilitation: Study Protocol for a Multicenter, Randomized Controlled Trial
Trial registration {2a and 2b}	The study protocol is registered on the German Clinical Trials Register (DRKS): DRKS00020373
Protocol version {3}	Date 19.10.2021 Version 3
Funding {4}	German Pension Insurance Central Germany, German Pension Insurance Berlin-Brandenburg
Author details {5a}	Puerto Valencia, Laura ^1^; Prof. Dr. Arampatzis, Diamantes ^2^,; Dr. Beck, Heidrun ^3^,; Prof. Dr. Dreinhöfer, Karsten ^4^;, Drießlein, David ^5;^,; Prof. Dr. Mau, Wilfried ^6^;, Zimmer, Julia-Marie^6^;, Dr. Schäfer, Michael^7^,; Dr. Steinfeldt Friedemann^8^; Prof. Dr. Wippert, Pia-Maria^1,9^ ^1^ Medical Sociology and Psychobiology, University of Potsdam, Potsdam, Germany ^2^ Department of Training and Movement Sciences, Humboldt-Universität zu Berlin, Berlin, Germany ^3^ University Center for Orthopaedics and Trauma Surgery, Hospital Carl Gustav Carus at Technical University Dresden, Dresden, Germany ^4^ Statistical Consulting Unit StaBLab, Ludwig-Maximilians-Universität München, Munich, Germany ^5^ Department of Orthopaedics and Trauma Surgery, Medical Park Berlin Humboldtmühle, Charité Berlin, Germany ^6^ Institute for Rehabilitation Medicine, Interdisciplinary Center for Health Sciences, Medical Faculty, Martin-Luther-University (MLU) Halle-Wittenberg, Halle, Germany ^7^ German Pension Insurance Berlin-Brandenburg, Rehabilitation Clinic Hohenelse, Department of Orthopedics, Rheinsberg, Germany ^8^ Johannesbad Specialist Clinic & Health Center Raupennest, Orthopedic Clinic in Altenberg, Germany ^9^ Faculty of Health Sciences Brandenburg, University of Potsdam, Potsdam, Germany
Name and contact information for the trial sponsor {5b}	Principal Investigator: Prof. Dr. Pia-Maria Wippert Professur für Medizinische Soziologie und Psychobiologie, Universität Potsdam, Am neuen Palais 10, 14469 Potsdam, Deutschland, wippert@uni-potsdam.de Trial sponsor and funding: German Pension Insurance Middle Germany (DRV), German Pension Insurance Berlin-Brandenburg (DRV)
Role of sponsor {5c}	The DRV is not involved in the design, writing of the report or the decision to submit the report for publication.

## Introduction

### Background and rationale {6a}

Chronic diseases are the main burden on the German healthcare system, and low back pain (LBP) is one of the most common ones [1]. Back pain is frequently associated with limited physical ability, sickness-related absence from work, and high socio-economic costs [2–4].

In Western Europe, low back pain was the highest contributor to disability (years lived with disability) and overall burden of disease (disability-adjusted life years) compared to 290 other conditions, according to the Global Burden of Disease 2010 Study [1]. In Germany 2017, LBP was also the leading cause of disability (years lived with disability) and the second most common cause of overall burden of disease (disability-adjusted life years) [2]. Considering 2019/2020 estimations, the 1-year prevalence of any type of back pain in Germany was 61%, while the lifetime prevalence of chronic back pain was 16% [5].

Moreover, the mean duration of incapacity for work in Germany is about 12.6 days. Back pain caused almost 6% of these days, being the second most important single medical diagnosis to contribute to incapacity [3]. Direct and indirect costs of back pain in Germany 2015 resulted in a cumulative estimation of approximately 4.5 billion (thousand million) euros (EUR) [4]. In the Berlin-Brandenburg region, a quarter of the days of incapacity for work are due to musculoskeletal disorders, lasting as long as 23 days [6]. The estimated loss of productivity per year in the region for about 20 days of incapacity for work rounds the 4.3 billion EUR [6].

Furthermore, psychosocial factors play a significant role in the development of chronic pain syndromes [7], including low back pain [8]. In line with the biopsychosocial model, LBP is better explained by the interaction between psychological, physical, and social factors than by the single anatomical or physiological model [9]. According to the national health care guidelines for the diagnosis and treatment of non-specific low back pain recommendations [10], an early screening of psychosocial risk factors is vital for an optimal therapy design (unimodal vs. multimodal). Until now, a lack of practical screening instruments for the early identification of individual needs regarding exercise interventions has been described. The recently published screening tool named Risk Stratification Index (RSI) [11] enables an estimation of the chronicity risk by means of psychosocial factors, supplemented by the Risk Prevention Index (RPI-S). The RPI-S additionally allows the identification of risk components in four psychosocial areas (pain experience, stress, medical care context, care context) [11]. Both screening tools aim to inform about the individual need for further psychosocial services and if specific supplementary training, physiotherapy, and/or psychosocial components are needed in order to increase intervention efficiency [12].

In rehabilitation ambulatory care, a multidisciplinary approach combining medical, physiotherapeutic, and psychological fields has already been introduced in Germany. However, its application has been challenging in diverse areas, and individualization of the therapy is still missing [10]. Its implementation has not reached most rural areas [10] and has to date been limited only to rehabilitation clinics and centers [13]. More often, no rehabilitation center is located in areas with low infrastructure; hence, the offer is missing in around 60% of the cases in Germany [10, 13, 14]. Until now, aftercare measures have been underused or were not easily accessible [14]. To our knowledge, no offer of an individualized home-based rehabilitation program is available. It is therefore of the greatest interest to assess these programs in the context of rehabilitative measures.

### Objectives {7}

This is a study on rehabilitation aftercare for back pain patients.

The aim of the study is to evaluate whether and how well an individually tailored rehabilitation aftercare program for back pain patients works. The following hypotheses are going to be examined:

1. An individualized and home-based aftercare program is more effective than regular outpatient rehabilitation aftercare in terms of pain reduction.
2. An individualized and home-based aftercare program is more effective than regular outpatient rehabilitation aftercare in terms of work ability.
3. An individualized and home-based rehabilitation aftercare leads to better patient compliance and more sustained intervention effects than regular outpatient rehabilitation aftercare.
4. An individualized and home-based rehabilitation aftercare could be implemented and incorporated long term (feasibility) in the regular rehabilitation aftercare routines of the German Pension Insurance.

### Trial design {8}

**Fig. 1:**
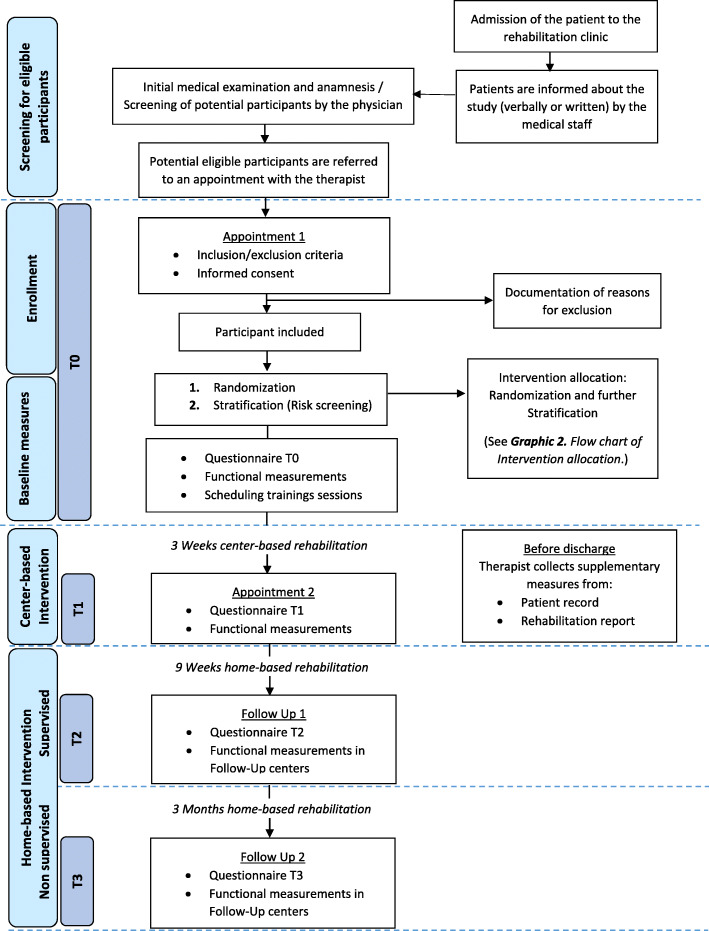
Flow chart of the study procedure

**Fig. 2:**
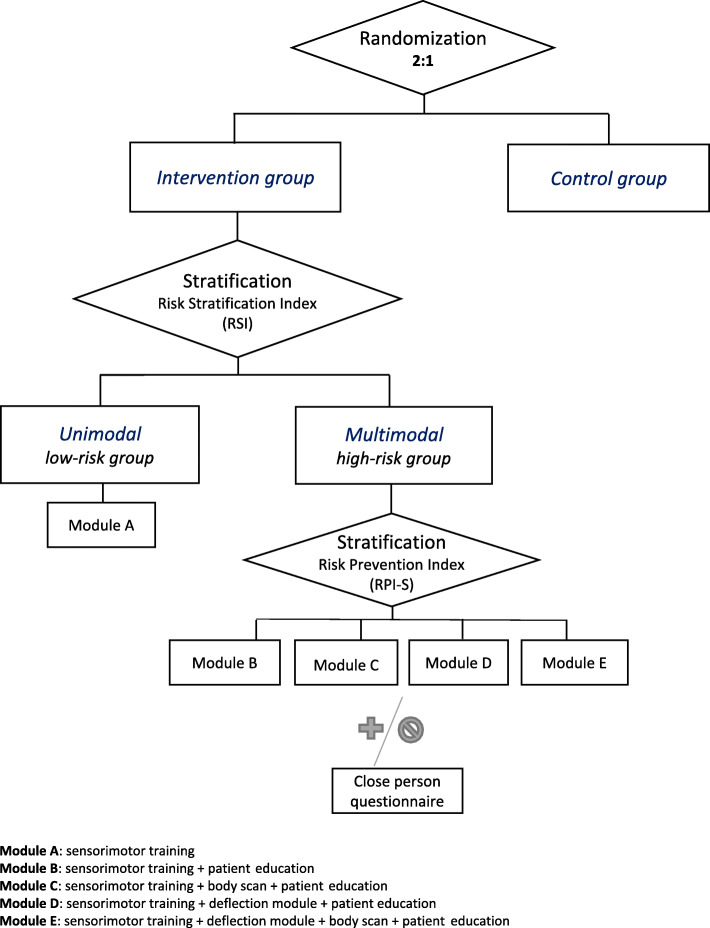
Flow chart of the intervention allocation

**Fig. 3:**
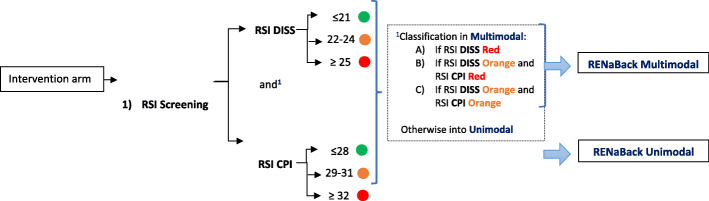
Flow chart of the Risk Stratification Index (RSI) allocation procedure into the unimodal or multimodal arm

**Fig. 4:**
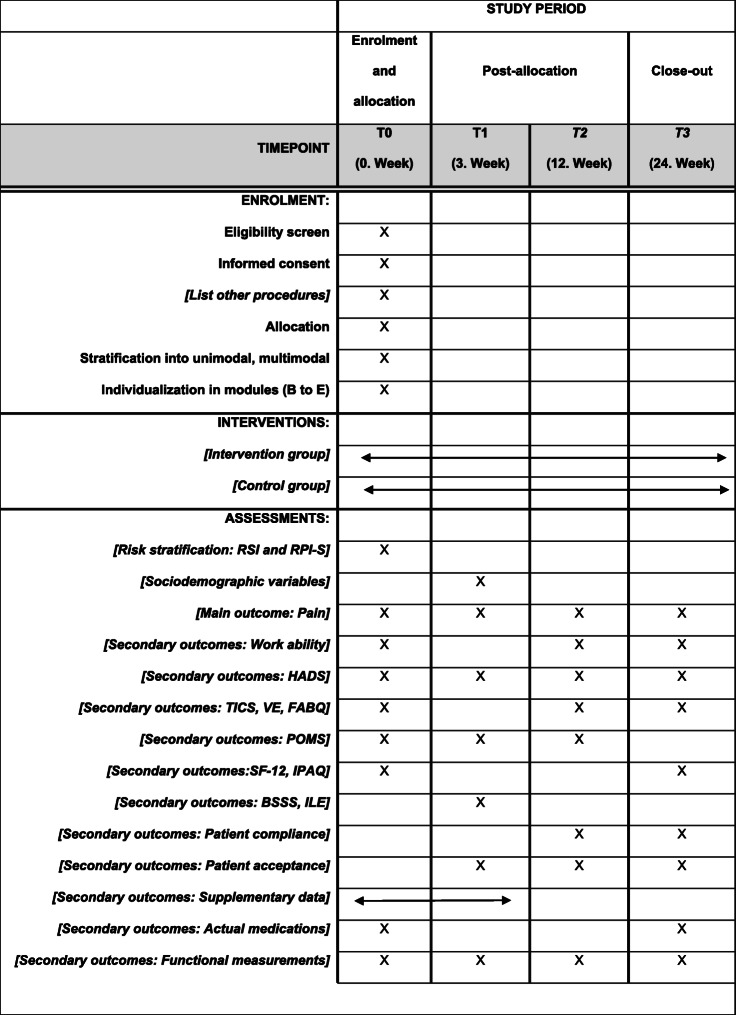
Schedule of enrollment, interventions, and assessments

Multicenter intervention trial: A randomized controlled prospective longitudinal intervention study (RCT) will be conducted at several hospitals in the regions of Berlin-Brandenburg and Central Germany. The study includes a total of four measurement points: at rehabilitation clinic admission (t0: baseline), at the end of the rehabilitation stay (t1), at the end of the 9-week home training phase (t2), and 3 months after the end of the accompanied training (t3: follow-up). Questionnaires and functional and clinical data are collected. After signing the informed consent, patients will be randomly allocated to the intervention group (including 2 subgroups arms; unimodal and multimodal intervention) or the control group (treatment as usual) using a 2:1 ratio. The subgroup allocation within the intervention group, either to a unimodal or a multimodal therapy will follow the Risk Stratification Index (RSI) [11]. An additional assignation to modules within the multimodal group will follow the Risk Prevention Index (RPI-S) [11] (see Figs. 1, 2, and 3.). The initial random 2:1 ratio allocation should ensure enough patients in each subgroup arm (unimodal or multimodal) to be able to analyze the differences between both and confirm the discrimination’s ability of the screening tool in this population (see Fig. 4). The framework of the trial is superiority.

Status: currently recruiting and in follow-up.

Dates of recruitment: 21.09.2020 to 31.12.2021. Dates of follow-up: 28.01.2021 to 17.06.2022

## Methods: participants, interventions, and outcomes

### Study setting {9}

Six rehabilitation clinics in the regions Berlin-Brandenburg and Central Germany participate in the study.

• Medical Center “Oberlin Rehaklinik Hoher Fläming” in Bad Belzig

• Medical Center “Rehabilitationsklinik Lautergrund, Bad Staffelstein” in Schwabthal

• Medical Center “Johannesbad Fachklinik & Gesundheitszentrum Raupennest” in Altenberg

• Medical Center “Rehabilitationsklinik Hohenelse” in Rheinsberg

• Medical Center “Rehabilitationsklinik Göhren” in Göhren

• Medical Center “Medical Park Humboldtmühle” in Berlin

### Eligibility criteria {10}

Inpatients with back problems (ICD-10 diagnosis M50-54), 25 to 60 years old, admitted to one of the six rehabilitation clinics in Germany will be included.

Patients who had a surgery less than 6 months ago, have an acute infection or cold, or are pregnant will be excluded. Patients with an inflammatory rheumatic disease, severe neurological disease (e.g., dementia ICD-10 F00-F03, Alzheimer’s disease, epilepsy), severe psychiatric illness (e.g., schizophrenia ICD-10 F20, dysthymia F34.1), emotionally unstable personality disorders such as borderline disorders (ICD-10 F60.3x), intellectual limitations (such as ICD-10 F70 to F79), and diseases which contraindicate physical activity or participants who are unable to stand on one leg will be also excluded. Furthermore, limited knowledge of the German language is an exclusion criterion. (The trial targets employed patients who could return to work).

### Who will take informed consent? {26a}

After admission to the rehabilitation clinic, the patient will be contacted either by the corresponding physician (ward or head physician) or other healthcare workers (nurse, therapist, or scheduler). The first member to contact the patient will inform him/her about the study and hand the flyer with the written information. Furthermore, the physician conducting the initial clinical examination will decide whether the patient is eligible for the study. If eligible, an appointment with the study therapist will be scheduled. The therapist will then explain the details of the study and take the informed consent of the patients who agree to participate.

### Additional consent provisions for the collection and use of participant data and biological specimens {26b}

Not applicable. The informed consent includes the provision for the collection of all participant data stated in the protocol. No biological specimens are contemplated at the time of this protocol.

## Interventions

### Explanation for the choice of comparators {6b}

The intervention group will receive a home-based exercise program after an initial center-based training. Currently, in Germany, there is no offer of a home-based program, which would allow patients a soft transition from the rehabilitation clinic to their everyday life. Likewise, rural areas have difficulty accessing the programs implemented exclusively in rehabilitation centers and clinics [13, 15].

In line with recent studies, diverse therapy modules tailored to individual patient needs, when based on an early screening for psychosocial risk factors, enable an optimal therapy for chronic back pain [10, 16, 17]. Accordingly, an initial differentiation between low-risk (unimodal) and high-risk (multimodal) groups through the RSI score, followed by an additional module individualization by the RPI-S score, is encouraged [11].

The intervention incorporates behavioral therapy modules together with exercise in the multimodal group. A combined approach of sensorimotor training, deflection, and body scan targets neurobiological adaptation processes. Furthermore, psychoeducation involving caregivers increases compliance and dismantles unfavorable support structures in the social environment [17].

The control group will receive the current rehabilitation aftercare offered by the German pension insurance “DRV” in the rehabilitation centers. It is relevant and appropriate to compare the new program with the “treatment as usual” program already implemented.

### Intervention description {11a}

#### Intervention group

The intervention has a total duration of 12 weeks. During the first 3 weeks, the training takes place within the clinic, three times per week, 30 min per session (3 weeks supervised center-based). In the following 9 weeks, the participant continues the training program independently at home with the help of training materials (diary, videos, mat, and block). In case of questions, the study therapist can be contacted via telephone during the 9-week home-based intervention.

- Intervention arm I (unimodal): The low-risk group (unimodal) receives sensorimotor training exclusively. The Sensorimotor Training Program (SMT) consists of four exercises (quadrupedal/all-fours stability, deadlift/rowing, double leg–single leg heel-pad stance, and side planks) performed in three sets of ten repetitions each. All exercises train the muscles surrounding the torso, two exercises initiate this training by an impulse via the extremities. The program contains 12 ascending levels of difficulty to enable individualized and adaptive use. At the beginning, the study therapist determines participants’ entry level depending on the individual training condition. Ideally, the level of difficulty would increase by one level weekly through the use of additional loads and unstable material. If not possible, the training will continue for another week at the existing difficulty level. More details on the sensorimotor training program are provided in Table 1 [15].

**Table 1:** Sensorimotor training program (SMT) exercises levels 1 to 12

Exercise 1: quadrupedal/all-fours stability		Exercise 2: deadlift/rowing		Exercise 3: double leg–single leg heel-pad stance		Exercise 4: side planks	
Stable ground	Unstable ground	Stable ground	Unstable ground	Stable ground	Unstable ground	Stable ground	Unstable ground
1. Hand and knee stance: cat’s hump/horse’s back 2. Hand and knee stance diagonal arm and leg: from body center upwards (horizontal) 4. Hand and feet stance: bending, stretching a leg 6. Hand and feet stance: release arm, trunk rotation	3. Hand and knee stance diagonal arm and leg: from body center upwards (horizontal) 5. Hand and feet stance: bending, stretching a leg 7. Hand and feet stance: release arm, trunk rotation 8. Planks: leg horizontal 9. Planks: diagonal leave arm and leg 10. Planks: leave arm, rotate the trunk 11. Planks: leave arm and diagonal leg, rotate trunk 12. Press-up: leave arm	1. Rowing plus additional weight 3. Rowing in ball stance plus additional weight 5. One-handed rowing plus additional weight 6. One handed rowing plus additional weight in ball stance 9. One-handed rowing plus additional weight in single-leg stance 10. One-handed rowing plus additional weight in single leg ball stance	2. Rowing plus additional weight 4. Rowing in ball stance plus additional weight 7. One-handed rowing plus additional weight 8. One-handed rowing plus additional weight in ball stance 11. One-handed rowing plus additional weight in single-leg stance 12. One-handed rowing plus additional weight in single-leg ball stance	1. Bipedal: heel-pad stance 3. Unipedal stance plus hip abduction 4. Unipedal stance plus hip abduction and leg extension 6. Unipedal ball stance plus hip abduction and leg extension 10. Unipedal squat 11. Unipedal squat plus additional weight	2. Bipedal: heel-pad stance 5. Unipedal stance plus hip abduction and leg extension 7. Unipedal ball stance plus hip abduction and leg extension 8. Squat in ball stance 9. Squat in ball stance and hip bending 12. Unipedal squat plus additional weight	1. Knee on the ground: hip up/down 3. Knee on the ground: hip released from the ground and hold 5. Knee on the ground: hip up/down without putting down the pelvis 7. Legs stretched, hip fixed upwards 10. Legs stretched, release leg from the ground 12. Legs stretched, release leg and diagonal arm from the ground: horizontal contact	2. Knee on the ground: hip up/down 4. Knee on the ground; hip released from the ground 6. Knee on the ground: hip up/down without putting down the pelvis 8. Legs stretched, hip fixed upwards 9. Legs stretched, hip up/down 11. Legs stretched, release leg from the ground

Source: Adapted from Niederer D, Vogt L, Wippert P-M, Puschmann A-K, Pfeifer A-C, Schiltenwolf M, et al. Medicine in spine exercise (MiSpEx) for nonspecific low back pain patients: study protocol for a multicentre, single-blind randomized controlled trial. Trials. 2016;17 [1]:507

Interventional exercises details. For each exercise, level [1–12], surface (stable/instable), and description are provided; p. 6
- Intervention arm II (multimodal): The RPI-S score gives information about the type of individualized care required for patients in the high-risk group (multimodal). Along with the SMT, the multimodal group receives supplementary behavioral therapy modules. Multimodal intervention incorporates three behavioral therapy modules (BT)**.** The modules are patient education, cognitive distraction techniques, and the “body scan” (element from the Mindfulness-Based Stress Reduction Program [18]). Depending on their RPI-S risk profile, participants receive one of the following intervention module combinations:

1. Sensorimotor training + patient education
2. Sensorimotor training + body scan + patient education
3. Sensorimotor training + distraction module + patient education
4. Sensorimotor training + distraction  module + body scan + patient education

Additionally, patients could receive a questionnaire for a person they feel close to, mainly in order to give them support during the implementation of aftercare exercises.

##### Patient education

A multipart film (four parts, duration of more than 2 h) conveys knowledge on the topic of general pain, development and maintenance of pain, active pain control, pain regulation, and how to deal with everyday life pain incompatible processes. The patient education film also includes strategies for self-empowerment in pain management through interactive reflective exercises, exchange with the partner, and active approaches. It will be delivered to all patients allocated to the multimodal group at the beginning of the intervention.

##### Cognitive distraction

The cognitive distraction module was adapted to the various sensorimotor training exercises and proceeds parallel to the physical training [19]. The cognitive distraction task consists of the n-back task (memory working task, once with numbers and once with colors) and a word list assignment [20–22]. Adjustment of the level of difficulty according to the individual’s ability and needs is also available. The study therapist indicates at the beginning that one movement corresponds to one word or digit heard. Ten movements take place with a 30-s break between the individual sets of each exercise. At the start and end of the training set, a signal announces the movement execution.

##### Body scan (element from Mindfulness-Based Stress Reduction Program)

Four versions of body scans based on the “Mindfulness-Based Stress Reduction” Program [18, 23–25] together with an introduction about mindfulness constitute one of the behavioral therapy modules. The body scans are presented on the DVD invariably after the sensorimotor training exercises.

At the beginning, the therapist advises patients on the practice of mindfulness exercises and introduces them to the first body scan. The therapist points out the importance of continuous regular practice and patience, guiding the patient to listen to it in either a sitting or lying position. Further they give the patient sufficient time in a quiet warm atmosphere, allowing a comfortable and safe starting position. The body scans comprise a longer version (Nr.1), a shorter one, and different levels of difficulty as the program progress (Nr. 2 to 4).

##### Close person support in the implementation of aftercare exercises and questionnaire

Multimodal patients with RPI-S high-risk scores in the domains “social environment” and/or “medical care environment” will be informed about the close person questionnaire, which is designed and conducted by the MLU Halle-Wittenberg. If the participant agrees, at T0, the study material will be sent in a pre-stamped envelope to a person they feel close to (usually a spouse, partner, child, or best friend). The close person receives information about the study, the declaration of informed consent, the questionnaire, and information on how to support the patient regarding physical activity. If they agree to participate, the completed questionnaire and one copy of the declaration of informed consent in a pre-stamped envelope will be sent to the MLU Halle-Wittenberg. At T2, a follow-up questionnaire will be sent to the same close person.

The questionnaire (sent at T0 and T2) is based on a previous study [26]. It incorporates items regarding given support (of physical activity/aftercare exercises) and social distress from the Berlin Social Support Scale  [27] and the social support scales for illness (SSUK) [28]. Likewise, it includes resources, barriers, and the importance of physical activity, as well as the age, gender, and health status of the close person.

#### Control group

Patients in the control group receive the standard aftercare treatment for back pain in rehabilitation centers (“treatment as usual”). This comprises the “T-RENA,” “IRENA,” and “Reha-Sport” programs of Germany.

T-RENA is a unimodal rehabilitation aftercare program suitable for persons with impairments or functional limitations on the postural and locomotor system. It is a training supported by the use of equipment carried out in groups of up to 12 people, usually containing 26 units of 60 min each. T-RENA includes preparatory exercises for subsequent muscle building training (e.g., general endurance training, general strength training), mobility, coordination, strength, and endurance. Moreover, it aims to develop and consolidate basic motor patterns together with daily life activities, also training on compensation techniques [29].

IRENA refers to a multimodal aftercare group service for 10–12 participants. It includes up to 24 units lasting at least 90 min each. IRENA is indicated when there are persistent functional and/or cognitive limitations, the need of lifestyle stabilization, and behavioral changes, further when support on specific workplace and professional reintegration problems is required and in the transfer of skills learned at rehabilitation. Therapy is provided in at least two of the following listed services: sports and exercise therapy (endurance or muscle-building training), physiotherapy (including spinal gymnastics), motivation guidance (promotion of behavioral changes), nutrition (nutritional counseling), psychology (stress management, relaxation training, problem-oriented group work), and social work (including professional orientation). In the case of orthopedic diseases (such as chronic back pain), the services offered usually include physiotherapy, sports and exercise therapy, and group psychological work for pain management [29].

Reha-Sport consists of gymnastics, athletics, swimming, and group exercise games, which are carried out in fixed groups with a trainer. It does not include equipment training, and the duration of a unit is at least 45 min [29].

The focus of these three aftercare rehabilitation programs (T-RENA, IRENA, and Reha-Sport) is on physiotherapy, movement, and behavioral therapy modules; however, they lack personalization of therapy content regarding individual needs. Furthermore, all three are conducted in an ambulatory setting and follow the routine of the German Pension Insurance aftercare program.

At the end of the rehabilitation at the clinic, one or none of the aftercare programs is recommended [29]. If the patient is unable to attend the rehabilitation center, the patient does not receive any aftercare treatment.

### Criteria for discontinuing or modifying allocated interventions {11b}

In the case of any adverse event (AE) or serious adverse event (SAE), the study therapist immediately informs the physician (from the rehabilitation center at T0 and T1, or from the follow-up center at T2 and T3). The review of the situation and decision to stop the intervention will be taken according to the physician’s judgment. Within 1 week, the study therapist will inform the study coordinator as well in order to document the event.

In the study, an adverse event is classified as follows:

- Unintentional illness or injury
- Adverse clinical observation (including abnormal laboratory results)

A serious adverse event is defined as a health impairment that:

- Leads to death (during the study or up to four weeks after the end of the study)
- Is life-threatening
- Leads to a permanent or temporarily significant disability/impairment
- Leads to hospital admission (> 24 h) or extension of an existing hospital stay
- Is a congenital anomaly or birth defect (in descendants of patients)

Beyond, the patient can withdraw their informed consent at any time resulting in discontinuation of intervention. Furthermore, the change of randomized allocated intervention at T0 (from intervention to control group and vice versa) is exclusively possible as a result of an explicit patient request, and only after a detailed conversation about the benefits of the assigned intervention. Moreover, control group patients are offered access to a part of study materials, after completing their participation.

### Strategies to improve adherence to interventions {11c}

One of the strategies to improve adherence to the interventions is to schedule training sessions by assigning electronic appointments. This strategy supports the “follow-up adherence,” which refers to the completion of the scheduled assessment measures [30]. The procedure was already successfully tested and implemented in the pilot study (material unpublished), and it will be achieved in the study again with the help of the scheduling department of the rehabilitation clinic.

The study therapist is at the clinic at least 3 days per week and once after the discharge of the last participant. He/she is also available by telephone at fixed times during the first 12 weeks of intervention to support all participants. This strategy intends to reduce misunderstandings about training regimens [30].

Moreover, at the first session, participants in the intervention group receive a training journal and an exercise booklet to improve the adherence to the intervention. Self-reporting diaries are a common measure of adherence in clinical trials [31]. Besides, the training journal is used to monitor adherence and the participant will return it at the end of the follow-up home-based training (T3).

### Relevant concomitant care permitted or prohibited during the trial {11d}

Concomitant care is allowed during the trial.

The trial setting is the rehabilitation clinic and home. Therefore, no broader supervision is possible or intended. The study has a pragmatic approach with the aim of allowing the patient to function in his or her real context. At T0 and T3, the actual medications (prescribed or not prescribed) will be recorded.

### Provisions for post-trial care {30}

No post-trial care is planned.

### Outcomes {12}

#### Risk stratification

##### Risk Stratification Index (RSI) and Risk Prevention Index (RPI-S)

The Risk Stratification Index (RSI) is a screening tool that allows an estimation of the risk of low back pain chronicity (1-year prognosis) through psychosocial risk factors. It includes baseline pain, unhappiness, social support and status, distress (chronic worries), work dissatisfaction, misfortune, pain persistence, sleep problems, and other health care issues such as medication history and insurance status [11].

The Risk Prevention Index (RPI-S) is a screening tool that identifies further individual therapy needs of low back patients, based on the risk within four psychosocial domains (distress, pain experience, social environment, and medical care environment). It also incorporates expectations to exercise treatments [11].

Both screening tools were derived through LASSO (least absolute shrinkage and selection operator) models, which covered yellow, black, and blue flag factors for chronic back pain and disability, as well as demographic and protective factors [32].

#### Primary outcomes

##### Chronic pain

The study has 2 co-primary outcomes (Characteristic Pain Intensity and Disability Score).

*Chronic pain* is assessed by the German version of the Chronic Pain Grade Questionnaire (CPG) [33, 34], which comprises two subscales: characteristic pain intensity (CPI: 0 = “no pain” to 100 = “pain as bad could be”) and disability score (DISS: 0 = “no disability” to 100 = “unable to carry on any activities”) from the past 3 months. Both subscales consist of the mean of three individual Numeric Rating Scales ranging from 0 to 10, showing good internal consistency (Cronbach’s alpha 0.88 and 0.68, respectively, from the literature [34]). Furthermore, these subscales display moderate to high relations to other instruments measuring a patient’s disability. Therefore, the CPG questionnaire is a reliable and valid instrument when an uncomplicated grading of chronic pain severity is required [34]. The CPG is administered at all time points (T0, T1, T2, and T3). The main time point is T2.

#### Secondary outcomes

##### Current pain

Current pain intensity is also assessed before and after functional diagnostics at all time points by the *Numeric Rating Scale* (NRS), which is an 11-point numeric scale ranging from 0 (representing one pain extreme “no pain”) to 10 (representing the other pain extreme “worst pain imaginable”). High test-retest reliability in patients with arthritis has been shown, reporting a Pearson *r* coefficient of 0.96 before and after medical care [35].

The *Brief Pain Inventory* [BPI] [36] includes two subscales: pain severity and pain interference. The first one is the mean of four current pain items (worst, least, average, and now), while  the second one is constructed by the mean of pain interference during seven daily activities (general activity, walking, work, mood, enjoyment of life, relations with others, and sleep) [36]. Cronbach’s alpha is reported to be 0.88 for pain severity and 0.92 for pain interference [37].

##### Functional and work ability

This is defined as work ability, days of incapacity to work, and subjective prognosis of employment.

*Work ability* is measured via the Work Ability Index (WAI) following the 2017 WAI Germany network guideline [38]. This questionnaire comprises seven thematic dimensions. Counting all dimensions, the highest score 49 indicates maximum work ability, while the lowest 7 suggests the opposite. The first dimension assesses the current work ability compared to the best work ability ever accomplished. The second one measures work ability in relation to work demands. The third and fourth dimensions record current illnesses diagnosed and their related impairment on work performance. The fifth dimension collects the number of sick days in the past year (days of incapacity to work). The sixth assesses the ability to work 2 years from now. Finally, the last dimension asks about the mental performance reserves [39]. Cronbach’s alpha (reliability) reported in the literature is 0.83 [40].

*Days of incapacity* to work are recorded in one of the WAI questions (“How many full days did you stay away from work due to a health problem (illness, health care or examination) in the last year (12 months)?”), and it has five categorical answers from none to within 100 and 365 days. Moreover, the rehabilitation report from the study center could confirm this information (days of incapacity to work).

*Subjective prognosis of employment* is characterized by the total score of the subjective prognosis of gainful employment (SPE) questionnaire. The score ranges from 0 to 3, the higher score means a worse prognosis. Internal consistency was confirmed in a cohort study of patients with severe low back pain and is recommended for rehabilitation research [41].

##### Psychometric items

Questions about health-related quality of life (12-Item Short-Form Health Survey: SF-12) [42]; physical activity (International Physical Activity Questionnaire (IPAQ) [43]); subjective well-being regarding stress, moods, fatigue, anxiety, and depression (Trier Inventory for Chronic Stress (TICS) [44], Profile of Mood States (POMS) [45], vital exhaustion (VE) [46], Hospital Anxiety and Depression Scale (HADS) [47]); fear-avoidance beliefs (FABQ-D) [48]; support (Berlin Social Support Scales (BSSS) [27]); and life-changing events (ILE) [49] are recorded.

*SF-12 short form of the Health Survey SF-36* [42]: the questionnaire measures non-disease-specific quality of life. Both scales have values from 0 to 100, higher values indicate a better health-related quality of life. Cronbach’s alpha range from 0.72 to 0.89 [42].

*The International Physical Activity Questionnaire Short Form* [IPAQ] [43]: it measures specific types of physical activity among adults: walking, moderate-intensity, and vigorous-intensity activities. The total score (median and interquartile range) combines the duration and frequency of the physical activity weighted by its energy requirement [50]. Cronbach’s alpha is 0.60 for this short version [51].

The *Trier Inventory for Chronic Stress* [TICS] [44]: the questionnaire encompasses a 3-month observation period. It comprises 57 items with five answers from 0 (“never”) to 4 (“very often”). Ten scales characterize different areas of chronic stress. Workload, social overload, and pressure to succeed scales describe stress related to excessive demands. The scales work dissatisfaction, excessive demands at work, lack of social recognition, social tension, and social isolation measure stress connected to a lack of needs satisfaction. Furthermore, the chronic concern and screening scales serve as a global measure of chronic stress. A Cronbach’s alpha between 0.84 and 0.91 is known from the literature [44].

*Profile of Mood States* [POMS] Short Form, German version [45]: it measures psychological well-being (mood) and includes four scales (depression/anxiety, fatigue, vigor, and irritability) from 35 individual items. Cronbach’s alpha ranges between 0.89 and 0.95 [45].

*Vital exhaustion* [VE] [46]: VE is a measure using a nine-item short-form German version of the original 21-item Maastricht VE Questionnaire. The items cover excessive fatigue, sleep problems (falling, waking up at night or unrested),  general discomfort, apathy, irritability, loss of energy, and demoralization; 18 is the highest score indicating severe exhaustion; furthermore, the Pearson *r* coefficient reported is 0.94 [46].

*Hospital Anxiety and Depression Scale* [HADS] [47]: this questionnaire assesses anxiety and depression symptom frequency and severity within the last week. It consists of 14 items building the two scales anxiety and depression. Both scales range from 0 to 21, have good validity, and report a Cronbach’s alpha of 0.80–0.81 [47].

*Fear Avoidance Beliefs Questionnaire* [FABQ-D], German version [48]: it consists of 16 items building 3 subscales (physical activity, work-related, and work prognosis) ranging from 0 to 30 [48]. A study on neck pain patients reports a Cronbach’s alpha of 0.90 [52].

*Berlin Social Support Scale* [BSSS] [27]: the questionnaire comprises six subscales regarding cognitive and behavioral social support aspects. Along, two main scales (perceived available and actually received social support) can be built. For both main scales, the internal consistency Cronbach’s alpha is 0.83 [27].

*Inventory of Life-Changing Events* [ILE] [49]: a total of 40 life-changing events are listed, in which participants can indicate whether they had already experienced such event and how often during the last 2 years. The number of a person’s life-changing events is analyzed as an accumulated number. In addition, in three of these events, the participant can indicate the subjective burden of the event by means of 10 extra questions. This score range from 0 to 48 points, a higher number means a higher burden. In a reliability study about the correlations between life events and diagnosed diseases, the instrument was judged as valid [49].

##### Patient compliance and acceptance

To measure patient compliance to the intervention program (therapy adherence), the frequency of sensorimotor training, patient education activities, and the use of body scan is registered in the training journal. This frequency may range from zero to three times weekly during 24 weeks. Further, the level of sensorimotor training difficulty is recorded as well as the degree of pain before and after exercises, using the 11-point *Numeric Rating Scale*. Additionally, a self-designed question “How often do you perform the aftercare exercises?” will be asked to all participants at T2 and T3 in order to have a subjective assessment of patient compliance.

The level of acceptance of the program will be assessed using self-developed question blocks (blocks represent the constructs defined through a factor analysis in the pilot study). The constructs are satisfaction with the program (it includes the questions “How satisfied are you with your aftercare program?” and “How do you rate the effect of the program you have completed?” from T2 and T3, Likert scale 0–10), self-competence acquired to continue with the program after the center-based rehabilitation stay (it consists of 8 questions from T1 such as: “How convinced are you that you will be able to use the learned exercises after the rehabilitation stay?”, “How well prepared do you feel with regard to time after rehabilitation stay?” and “How helpful did you find the preparatory exercises during rehabilitation stay?”, Likert scale 0–10), and sustainability (including “How confident are you that you will continue to use your aftercare program regularly?” from T2 and T3, Likert scale 0–10, and open questions as “What could stop you from continuing the exercises?”).

##### Supplementary data (clinical data)

Supplementary data to assess the initial health status and later intervention success include documented clinical data from the initial medical examination (regular orthopedic examination) and patient records which comprise disease duration, medication, basic physiological status (e.g., blood pressure, height, weight, blood lipids) as well as information from the rehabilitation report at discharge (complete details see Table 2).

**Table 2:** Supplementary measurements collected from clinical data

Patient record (initial medical examination, physiological status)	Rehabilitation report at discharge (from clinic)
• Anamnesis of the initial orthopedic examination	• Rehabilitation success
• Diagnosis of the initial orthopedic examination	• Degree of disability, level of care
• Duration of the disease and medical history	• Employment status, gradual reintegration
• Therapies to date (medicaments)	• Aftercare recommendation
• Change of medication during rehabilitation	• Benefits for vocational rehabilitation
• Height, weight, waist and hip size	• Ability to work on discharge
• Blood pressure, pulse	• Days of incapacity to work in the last 12 months
• Blood lipids (HDL, LDL, cholesterol, triglycerides)	
• Urine, liver, thyroid gland, blood count values	

In addition, a question directly to the participant about their actual medication “Are you taking any medications, prescribed or otherwise? If yes, please list the name, dose, and frequency” will be collected at T0 and T3.

##### Functional measurements (postural control, motion, and mobility)

Postural control is an important ability and an elementary requirement for the maintenance of balance and stability in static and dynamic processes. One of the most commonly used methods to evaluate postural competency is the quantitative measurement of the center of pressure (CoP). The CoP is the origin of all ground reactionary forces in the transverse plane. The CoP trajectory indirectly allows a quantification of postural competence, based on body sway in a quiet stance on a force plate [53, 54]. It has been shown that portable force plates can be an alternative to conventional force plates [55].

Postural control will be assessed through center of pressure (COP) measures on a balance board system. Therefore, one-legged measurements, two for the left and two for the right leg, and one two-legged, will be performed after one trial with a standing time of 60 s. Mistakes or the inability of standing for that time will be recorded. A sampling rate of 1 kHz and a resolution of 14 bit will be used.

Motion analysis includes measurements of trunk mobility, local dynamic stability of complex trunk movements, and core muscles and lumbar spine coordination.

Trunk mobility is determined by controlled bending of the trunk from an upright standing position. The lumbo-pelvic kinematics in the upright standing position and during forward trunk bending will be measured using two three-dimensional acceleration sensors (Biovision, Wehrheim; size 1 × 1 × 1 cm, 1000 Hz) attached to the skin at the level of thoracic vertebrae 12 (T_12_) and sacral vertebrae 1 (S_1_). The pelvic (Base_pelvic_) and trunk (Base_trunk_) angles in the upright stance will be calculated in the sagittal plane using the orientation of the local coordinate system of the attached sensors in S_1_ and T_12_, with respect to the global coordinate system.

Coordination of the pelvis and lumbar spine is determined by the ratio of lumbar spine rotation to pelvic rotation: lumbo-pelvic ratio (LPR) in the sagittal plane during maximum protrusion. The same test setup is used to measure the LPR as used to determine the range of motion of the trunk.

The range of motion (RoM) of the pelvis and trunk of the patients will be measured in the sagittal plane during a controlled maximum forward trunk bending. The lumbo-pelvic ratio (LPR) will be calculated as the ratio of the changes in lumbar spine orientation to the changes of pelvic orientation. LPR will be calculated for the whole forward bending motion (full) as well as for the first (early), second (middle), and final (late) third of the RoM.

The local dynamic stability of complex trunk movements is quantified by the method of non-linear time series analysis. For this purpose, 30 motion cycles of a three-dimensional trunk movement (kneeling) are used. The local dynamic stability of the trunk during a rhythmic pointing task will be investigated using the short-term Lyapunov exponent (sMLE) as a criterion for the assessment of the neuromuscular control of spine stability. The participants will perform a rhythmic pointing task alternating between the right and left hand in a kneeling position. A total of 30 cycles will be included in the time series analysis. We will measure the motion of the trunk using a three-dimensional (3D) acceleration sensor (Biovision, Wehrheim, Germany; size 1 × 1 × 1 cm, 2000 Hz) attached on the back at the T_2_ (2nd thoracic vertebra) level. The assessment of neuromuscular control will be evaluated using nonlinear time series analysis and reconstructing the state space of the trunk dynamics from the recorded data.

*Mobility tests* comprise the Timed Up and Go Test (TUG) and Chair Rising Test. Before and after the tests, current back pain intensity is recorded using the NRS (Numeric Rating Scale) from 0 to 10.

*Functional measurements* will be performed at all time points (T0/T1 in the rehabilitation center, and T2/T3 in follow-up centers).

### Participant timeline {13}

The main outcome of interest is pain measured as Characteristic Pain Intensity and Disability Score. The trial is divided into three phases: [1] 3 weeks stay in rehabilitation, [2] 9 weeks of supervised aftercare home training (training support by phone and digital media assistance), and [3] 12 weeks of independent aftercare home training. As shown in Fig. 4, measurements will take place at four time points in each group, at baseline (T0), directly before leaving the rehabilitation stay (T1), after 9 weeks of supervised aftercare home training (T2), and after 12 weeks of independent aftercare home training (T3).

### Sample size {14}

For the main study, the sample size calculation was based on a small effect size of *d* = 0.2 as well as assumptions on the normal distribution and variance equality. Alpha-error probability of 5%, beta-error probability of 20%, and a ratio allocation of 1:2 (control vs intervention) were considered. The repeated measures ANOVA for three time points between the groups with a correlation among repeated measures of 0.57 (results from pilot study, unpublished material, based on CPG Disability score) calculate a sample size of 843 (*n* = 562 in the intervention and *n* = 289 in the control group). Assuming a dropout rate of 30%, the total number of participants to be included in the study is *N* = 1204 (power analyses by G*Power; Faul, Erdfelder, Lang & Buchner, 2007) [56].

### Recruitment {15}

The first contact with the staff upon admission to the rehabilitation clinic is either through the corresponding physician (ward or chief physician) or by other medical staff (nurses, therapists, scheduling personnel). The personnel inform patients about the study orally and/or via an information flyer. Study participants receive individual feedback on their health profiles.

- Planned/actual: actual
- Date of first enrollment: 21.09.2020
- Target sample size: 1204
- Monocenter/multicenter trial: multicenter trial
- National/international: national

## Assignment of interventions: allocation

### Sequence generation {16a}

The method of generating the allocation sequence consists of computer-generated random numbers. A stratified randomization per center is conducted. Concretely, a block randomization with a fixed block size is planned for each center. After assigning a random number (generated by Excel) to a list of numbers (block size), the blocks are sorted by their random number, resulting in a random 2:1 assignment to the intervention or control group for each center at each time point.

### Concealment mechanism {16b}

At the beginning of the study, each center will receive a fixed-size randomization numbers list. Every 2 weeks or when needed, the study therapist will receive the list with additional randomization numbers.

### Implementation {16c}

The data-monitoring personnel at the university generate the allocation sequence. The enrollment of participants takes place in the rehabilitation centers, afterwards the study therapist assigns the interventions following the randomization results.

## Assignment of interventions: blinding

### Who will be blinded {17a}

Open trial, neither participants, intervention instructors, nor outcome assessors are blinded to the group assignment.

However, participants do not receive information about the study objectives, regarding whether they receive the novel intervention studied. Furthermore, the instructors do not inform participants about their risk classification and the meaning of the individualized intervention modules.

### Procedure for unblinding if needed {17b}

Does not apply

### Data collection and management

#### Plans for assessment and collection of outcomes {18a}

The reliability of the study instruments (questionnaires and functional measurements) was verified through a test-retest analysis in September 2020. The feasibility of the trial was confirmed by means of the 8-month pilot study conducted during 2019/2020 in one rehabilitation clinic in Germany.

The questionnaires are collected via the secure database portal ProWebDB®, whereby the data is forwarded directly to the security server of the University of Potsdam.

### Plans to promote participant retention and complete follow-up {18b}

After the in-person contact with the study therapist has ended (T1), the study therapist is available to the participants by telephone at fixed times during 12 weeks of the follow-up phase for any questions (until T2).

The participants are reminded by telephone at least 2–3 days before the T2 measurement to complete the online questionnaires sent by email. Two weeks before the follow-up examination at T3, the study therapist calls the test persons and reminds them of the interview.

The online access to the questionnaires (sent via e-mail) has a period of use between 14 and 21 days; afterwards, no data collection is possible. In any case, if it is not possible to complete the questionnaire online, paper questionnaires will be sent.

The close person survey also includes a reminder letter with the questionnaire at T2, in case of no response within two weeks.

### Data management {19}

The participants primarily answer the questionnaires in digital form using an online survey program (ProWebDB®). Data is collected centrally via an online system (ProWebDB®) that complies with the current General Data Protection Regulation of the European Union (DSGVO).

In the event of no internet access, computer problems, or other reasons, a copy of the questionnaire in paper is available to the study therapist. Questionnaires filled out on paper will be stored in a lockable cabinet at the university. Data on paper along with the training journal will be entered into the statistical software SPSS® by two employees, randomly checked, and followed by a plausibility check.

All the details concerning data management are stated in the statistical evaluation plan of the project.

### Confidentiality {27}

Potential participants interested in the study will be informed by the study personnel about the objectives, procedures, risks, data protection, etc. of the research project. Afterwards, they are given enough time to consider whether they wish to participate in the study. In case of voluntary participation, a declaration of consent must be signed. The written consent (informed consent) includes a declaration of consent for voluntary participation in the study, for (pseudonymized) data processing, and a release of medical confidentiality to authorize medical personnel (e.g., doctors or therapists) the disclosure of information for the study.

After consent for voluntary participation in the study, participants are assigned a specific pseudonymous code, which serves to ensure the pseudonymization of the data during the data collection period.

The original informed consent forms and the pseudonymous code sheets are collected by the study staff on site. These two documents are used to enter the name, code, address, telephone number, and survey status of study participants into a digital patient identification list. The pseudonymized code sheets will be irrevocably destroyed by the study staff as soon as the transfer to the patient identification list has been completed. The original informed consent forms are stored separately from the research data in a locked cabinet in the rehabilitation center data before being locked in the room cabinets at the university.

The patient identification list is confidential; password-protected, with restricted access only to the project manager; and stored separately from the research data on a security server of the university. If a study participant withdraws the previously given consent, they will be irrevocably deleted from the patient identification list.

Information about the traceability of personal data is only kept during the period of the study by the patient identification list. All data and documents that contain both personal data and coding together are irrevocably deleted after the completion of data collection. Since the patient identification list is irrevocably deleted immediately after the end of the data collection (after T3), a later assignment of the name of a person and the corresponding individual data is not possible.

Due to the early pseudonymization of the data at the first measurement time (T0), all data is only collected in a coded form. The study staff deals exclusively with coded data, which does not allow for individual conclusions about the study participants. All electronic data will be stored exclusively on the university security server at the end of the project. All pseudonymized coded research data will be stored at a security server.

### Plans for collection, laboratory evaluation, and storage of biological specimens for genetic or molecular analysis in this trial/future use {33}

If no basic physiological status information is recorded during the initial medical examination, the willingness to take a blood sample and store it for subsequent analysis is requested at the clinic check-in with the corresponding informed consent.

## Statistical methods

### Statistical methods for primary and secondary outcomes {20a}

Data is analyzed at the end after T3 data collection completion. Then, an examination of underlying assumptions for parametric or non-parametric hypothesis testing is applied. For the primary outcome pain (DISS, CPI), an analysis of variance (ANOVA) repeated measures for three time points (T0, T2, T3) between the groups (intervention vs control group) with covariates adjustment is planned. The covariates to include in the analysis are center, age, and sex. A complete case analysis is intended.

For secondary outcomes (psychometric items), missing value imputation follows only (in case of psychometric scores) the specific test manuals. Likewise, variance analytical testing is proposed. A sensitivity analysis is planned for concomitant therapy, such as medications.

### Interim analyses {21b}

Do not apply. Interim analysis is not contemplated through discussion with the founder and partners

### Methods for additional analyses (e.g., subgroup analyses) {20b}

We also plan a subgroup analysis comparing the multimodal and the unimodal group, with the control group. In these cases, using a repeated-measures ANOVA between the two groups mixed with covariates such as age, sex, and baseline pain is planned for each multimodal and unimodal group.

### Methods in analysis to handle protocol non-adherence and any statistical methods to handle missing data {20c}

Intention to treat analysis will be performed. Per-protocol analysis is going to be applied to compare the results in a sensitivity analysis.

### Plans to give access to the full protocol, participant level-data, and statistical code {31c}

Does not apply. We do not contemplate access to the full protocol besides their publication. The datasets generated for this study should be available in the future after completion of the trial [doi is requested at the University of Potsdam]. The statistical code will remain in the internal files of the responsible parties.

## Oversight and monitoring

### Composition of the coordinating center and trial steering committee {5d}

The coordinating center is the Department of Medical Sociology and Psychobiology at the University of Potsdam, Germany. The overall project leader is the professor of the department, who takes the main decisions and communicates with the German Pension Insurance. A research assistant (master’s degree) has the responsibility of project coordination, involved in the communication with the partners (universities and institutes) and monitoring at the clinics. Further research associates (post-doctoral degree and master’s degree) are responsible for the intervention and data management, supported by students’ assistants at the department.

### Composition of the data monitoring committee, its role, and reporting structure {21a}

Two independent people with a master’s degree (employed at the Department of Medical Sociology and Psychobiology at the University of Potsdam, but not involved in the project work) participate in the monitoring committee. For the initial phase (2 weeks before trial first enrollment), 1–2 days per week include intensive monitoring at the rehabilitation clinic. It comprises location characteristics and internal processes implementation. Additionally, the therapist’s education/training includes study aims, documentation, and intervention training.

After the first participant enrollment, a written weekly report about the recruitment process and general questions from the therapists is scheduled. An online meeting with the therapists one time per month is outlined, as well as personal visits to the clinic once every 3 months.

Once a week, online data collection is reviewed to identify possible coding problems. The monitoring committee meets once every 2 weeks, supervised by the department’s professor. The data monitoring committee operates and reports directly to the department’s professor. The funder of the study is not involved in these processes and does not influence data collection or analysis.

### Adverse event reporting and harms {22}

The contact persons in case of such an event are both the study therapist and the corresponding physician in the clinic during the center-based phase (T0–T1) or the physician in the respective follow-up center (T2–T3). If the study therapist has been informed first about the adverse event, he/she immediately informs the physician. It is always the treating physician who decides whether to exclude the patient from the study.

### Frequency and plans for auditing trial conduct {23}

Each study center will be monitored once every 3 months by the study coordinator of the coordinating center.

### Plans for communicating important protocol amendments to relevant parties (e.g., trial participants, ethical committees) {25}

Do not apply. A specific meeting to communicate amendments to parties is not planned. However, in the quarterly network meetings, a synopsis on important changes and the trial process is discussed.

### Dissemination plans {31a}

A final report to the German Pension Insurance is planned. If successful, this intervention could be implemented in the regular aftercare of medical rehabilitation in Germany. The publication of the trial results in a scientific journal is also intended.

## Discussion

The main purpose of the study is to compare the efficacy of an individualized home-based aftercare program to the regular outpatient care for the rehabilitation of back pain. For this purpose, a randomized controlled trial is performed. We expect to have improvements in terms of pain, work ability, patient compliance, and acceptance in our intervention compared to the standard program.

The study helps to ensure that particularly patients living far away from clinical rehabilitation centers receive individualized care.

Because the intervention consists of individualized programs (unimodal, multimodal, and their corresponding intervention modules) for different patient risks, we also expect to have a better response in other outcome measures such as health-related quality of life, subjective well-being, and sensation of pain. We will also explore whether changes in the outcomes occur within the intervention group (unimodal vs multimodal) and how these differences unfold over the follow-up measures. Regarding the comparison between these subgroups, the power of our study is here limited, since our sample size is only intended to compare the two randomized groups (main objective). Nonetheless, this study contributes to the research regarding integrative programs. The exercises and tasks of the program involved in the study are of value, since up to now, only limited data provide information on training individualization. Furthermore, implementation of psychosocial risk factors in low back pain research is limited [57].

One strength of the study is the relatively big sample size and the ability to include patients’ profiles from clinics in six different federation states in Germany. Accordingly, it will be possible to contemplate an external generalizability of the results in Germany. At the same time, the heterogeneity of the study population might limit the internal data interpretation. Sensitivity analyses are planned to consider the effect of covariates. Further, a common limitation of a behavioral intervention including exercises is the impossibility to blind the study staff delivering the intervention and participants to the group allocation; still, the participants are not aware of the study objective. Another limitation is our concealment mechanism: due to constraints of resource management, we are not able to send one randomization number each time, but a few randomization numbers all at once. This could allow a risk of selection bias, because at times, the therapist can predict the next participant group allocation.

The present study provides a feasible individualized program, accessible at home with low costs. One impact of this study is to provide more available rehabilitation aftercare programs to rural populations with different psychosocial risks.

### Trial status

Protocol version 3, Date 19.10.2021.

The study protocol is registered in the German Clinical Trials Register (DRKS) DRKS00020373.

Participant’s recruitment started on 21.09.2020 and is estimated to be completed by December 2022.

## Supplementary Information

**Additional file 1.**

## Data Availability

The datasets [generated] for this study should be available in the future, after completion of the trial [doi is requested at the University of Potsdam].

## Abbreviations

LBP: Low back pain
RSI: Risk Stratification Index
RPI-S: Risk Prevention Index
RCT: Randomized controlled trial
IRENA: Intensified Rehabilitation Aftercare Program
TRENA: Training Therapeutic Rehabilitation Aftercare Program
Reha-Sport: Rehabilitation Sport Program
ICD-10: International Classification of Diseases, Tenth Revision
DRV: German Pension Insurance
SMT: Sensorimotor training program
BT: Behavioral therapy modules
AE: Adverse event
SAE: Serious adverse event
T0…T3: Time points
CPG: Chronic Pain Grade Questionnaire (German version)
CPI: Characteristic pain intensity
WAI: Work Ability Index
SPE: Subjective prognosis of gainful employment
DISS: Disability Score
SF-12: 12-Item Short-Form Health Survey
IPAQ: International Physical Activity Questionnaire
BPI: Brief Pain Inventory
TICS: Trier Inventory for Chronic Stress
POMS: Profile of Mood States
VE: Vital exhaustion
HADS: Hospital Anxiety and Depression Scale (German version)
FABQ-D: Fear Avoidance Beliefs Questionnaire (German version)
BSSS: Berlin Social Support Scales
ILE: Life-changing events
CoP: Center of pressure
LPR: Lumbo-pelvic ratio
RoM: Range of motion
TUG: Timed Up and Go Test
DSGVO: General Data Protection Regulation of the European Union
DRKS: German Clinical Trials Register
MLU: Martin-Luther-University of Halle-Wittenberg
