# Supplementary material for: RENaBack: low back pain patients in rehabilitation—study protocol for a multicenter, randomized controlled trial
Source: Trials. 2021 Dec 18;22:932. doi: 10.1186/s13063-021-05823-3 (PMC8684145; doi:10.1186/s13063-021-05823-3)
Supplement: Supplementary file 1 — Additional file 1. [file 13063_2021_5823_MOESM1_ESM.docx]

Model consent form:
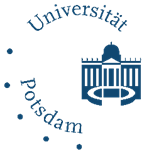


**Einverständniserklärung**

Titel der Studie:

**RENaBack – Stratifizierte Zuteilung zu multimodalen Interventionsbausteinen in der Nachsorge von Rückenschmerzpatienten**

Hiermit erkläre ich,

__________________________________ ___________________________

Vorname, Name geboren am

________________________________________________________________________________

Vollständige Anschrift

__________________________________ ___________________________

E-Mail-Adresse Telefonnummer

dass ich durch Herrn/ Frau _____________________________________________________________

mündlich und schriftlich über das Wesen, die Bedeutung, Tragweite und Risiken der wissenschaftlichen Untersuchung im Rahmen der o.g. Studie informiert wurde und ausreichend Gelegenheit hatte, meine Fragen hierzu in einem Gespräch mit der Studienleitung bzw. einer/m Studienmitarbeiter/in zu klären.

Ich habe die mir vorgelegte Studieninformation vom ___________ (Datum) verstanden und eine Ausfertigung der Studieninformation sowie dieser Einverständniserklärung erhalten.

Mir ist bekannt, dass ich mein Einverständnis jederzeit ohne Angabe von Gründen und ohne nachteilige Folgen für mich zurückziehen und einer Weiterverarbeitung meiner Daten jederzeit widersprechen sowie die Löschung bzw. Vernichtung meiner Daten verlangen kann.

Ich bin bereit, an der wissenschaftlichen Untersuchung im Rahmen der o.g. Studie teilzunehmen.

**Einverständniserklärung zur Datenerhebung- und Datenverarbeitung**

Ich bin damit einverstanden, dass bestimmte Kategorien meiner personenbezogenen Daten (Informationen zur Krankheitsgeschichte, psychisches Befinden, Schmerzerleben und körperliche Aktivität, funktionelle/ medizinische Routine-Daten wie in der Studieninformation beschreiben) durch Studienmitarbeiter erhoben und an der Universität Potsdam pseudonymisiert gespeichert werden.

**Ja □ Nein □**

Ich bin damit einverstanden, dass die Studienergebnisse in anonymer Form, die keinen Rückschluss auf meine Person zulassen, veröffentlicht werden (Fachzeitschriften).

**Ja □ Nein □**

Ich erkläre mich damit einverstanden, dass meine Daten (siehe oben) nach Beendigung oder Abbruch der Studie mindestens zehn Jahre aufbewahrt werden. Danach werden meine personenbezogenen Daten gelöscht, soweit nicht gesetzliche, satzungsmäßige oder vertragliche Aufbewahrungsfristen entgegenstehen (§ 35 Abs. 3 Nr. 1 BDSG).

**Ja □ Nein □**

**Datenweitergabe und Haftungsausschluss**

Mir ist bekannt, dass die erhobenen Daten nur zum Zwecke der Forschung im Rahmen der beschriebenen Studie verarbeitet werden.

Mir ist bekannt, dass die Studienteilnahme auf eigene Verantwortung erfolgt; es besteht kein Versicherungsschutz durch die Universität Potsdam.

**____________________________ _____________________________________________**

Ort, Datum Unterschrift des/der **Studienteilnehmers/in**
